# Adsorption-Induced Deformation in Microporous Kerogen by Hydrogen and Methane: Implications for Underground Hydrogen Storage

**DOI:** 10.1021/acs.langmuir.5c00197

**Published:** 2025-03-03

**Authors:** Saeed Babaei, Benoit Coasne, Mehdi Ostadhassan

**Affiliations:** † Civil Engineering Faculty, K. N. Toosi University of Technology, Tehran 1996715433, Iran; ‡ University Grenoble Alpes, CNRS, LIPhy, 38000 Grenoble, France; § Institut Laue Langevin, F-38042 Grenoble, France; ∥ Institute of Geosciences, Marine and Land Geomechanics and Geotectonics, Christian-Albrechts Universität, Kiel 24118, Germany

## Abstract

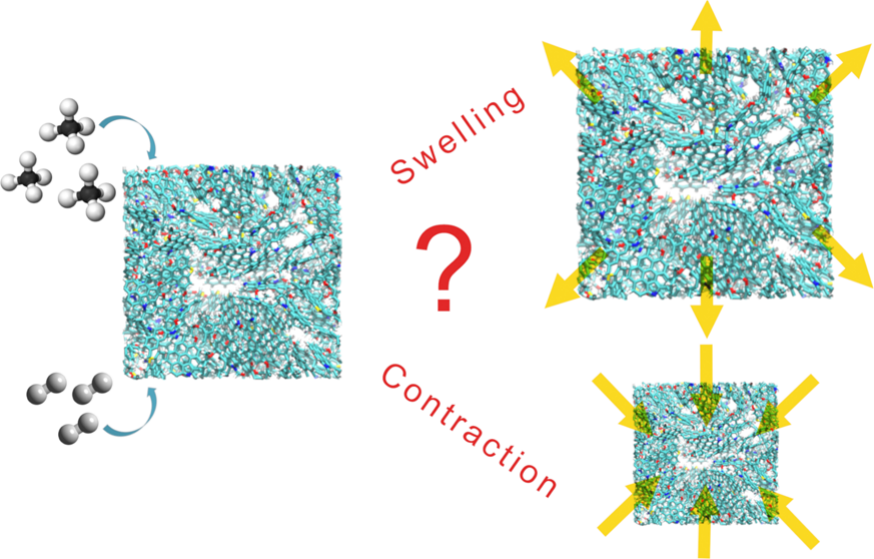

Accurately assessing the adsorption and diffusion behaviors of H_2_, CH_4_, and their mixtures are essential for estimating underground hydrogen storage (UHS). This understanding is critical for the safe and efficient storage of H_2_ in depleted shale gas reservoirs. Although H_2_ adsorption in kerogen has been extensively studied, adsorption-induced swelling remains unexplored in UHS. In this study, we investigate adsorption mechanisms using Lagrangian and Eulerian approaches and analyze diffusion in kerogen through molecular simulations. Our results reveal that in the presence of cushion gases like CH_4_, which exhibit stronger adsorption than H_2_, neglecting kerogen deformation can lead to an underestimation of storage capacity by approximately 40%. Furthermore, increasing pressure makes H_2_ adsorption behavior deviate from the consistent swelling trend that is observed with CH_4_, with kerogen either swelling or contracting depending on the pore size. Simulations also predict that H_2_ self-diffusion coefficient in porous kerogen is 1 order of magnitude higher than CH_4_. These findings highlight the importance of incorporating kerogen flexibility into the modeling of UHS involving multiple gas species to improve the accuracy and safety of H_2_ storage operations in shale reservoirs.

## Introduction

The shift from fossil fuels to renewable energy is vital for mitigating climate change.^1^ In this respect, hydrogen holds significant potential for reducing carbon emissions across industries, transportation, and power generation while enhancing energy security and decreasing reliance on imported oil and gas.^2^ Underground hydrogen storage (UHS) in geological formations has emerged as a key solution in the hydrogen economy. UHS allows for large-scale hydrogen storage during periods of low demand, ensuring retrieval when demand increases. It offers several advantages over above-ground storage, including large capacity, improved safety due to reduced risks of leak, and high-pressure storage that enhances energy density and suitability for fuel-cell applications.^3^

Shale is additionally emerging as a promising candidate for carbon capture, utilization, and storage (CCUS)^4^ and H_2_ storage^5^ due to its nanoporous structure and high surface-to-volume ratio, which facilitate gas adsorption and efficient desorption. Numerous experimental studies have examined H_2_ adsorption in clay^6,7^ and shale.^8^ However, these studies have predominantly been conducted at relatively low pressures. A key element of this process involves examining the adsorption and diffusive transport characteristics under realistic temperature and pressure conditions. The complex heterogeneity and nanoscale pore structures of subsurface systems present significant challenges in experimentally investigating gas behavior within porous rocks. Hence, molecular simulation is a valuable tool for analyzing gas properties at the atomic level to verify experimental data.^9−11^

Molecular simulations on shale are generally divided into inorganic and organic matter studies. Many of them^12−16^ have employed molecular simulations to examine H_2_ adsorption and diffusion in inorganic materials, such as clay, calcite and quartz, considering factors such as pressure, pore size, charge effects, and the presence of other gases. On the other hand, kerogen, the primary component of organic matter in shale, is insoluble in polar organic solvents^9^ and accounts for about 50% of the adsorption sites in shale gas.^17^ Research has shown that both CH_4_ and H_2_ preferentially adsorb at nitrogen and sulfur sites in kerogen,^18^ with CH_4_/H_2_ selectivity higher at low pressures, decreasing with pressure.^14,18,19^ Overmature kerogen exhibits greater H_2_ adsorption and more efficient desorption than immature kerogen^20,21^ while diffusivity decreases with pressure, with higher diffusion in slit pores that are not affected by thermal maturity.^22^

Despite advances, key questions in adsorption-induced deformations in microporous kerogen, a promising candidate for H_2_ storage due to its high surface area, are still unanswered. Traditional grand canonical Monte Carlo (GCMC) simulations assume a rigid kerogen matrix, justified by negligible swelling during H_2_ adsorption but overlooking potential structural deformations. Cushion gases such as CH_4_ are essential in UHS for maintaining reservoir pressure during H_2_ recovery;^6,14^ however, they induce kerogen deformation, alter adsorption capacity, and compete with H_2_ for adsorption, significantly affecting H_2_ recovery efficiency and purity, thereby underscoring the need for more accurate modeling. Recent hybrid approaches combining GCMC with molecular dynamics (MD) simulations in NPT^23−25^ or NVT^26−28^ ensembles can address these challenges, with the NVT method enabling internal matrix rearrangements during adsorption without net volume changes. Numerous studies combining GCMC and MD simulations have examined adsorption-induced deformations for gases such as methane,^24,26,28−32^ ethane,^27,30^ carbon dioxide,^24,30,31,33^ argon,^23^ and water^34−36^ in kerogen. The findings indicate that kerogen swelling directly correlates with the amount of gas adsorbed, with swelling decreasing as the adsorbate size increases.^26,30^ Notably, models that do not account for volume changes during swelling underestimate the adsorption capacity.^23^ Additionally, several MD studies^33,37,38^ have also investigated the role of kerogen deformation in transport, demonstrating that the associated adsorption-induced swelling phenomena might accelerate the diffusion of fluid molecules.

In addition to the significance of kerogen deformation, two fundamental frameworks are commonly employed to analyze swelling in adsorption phenomena: the Lagrangian and Eulerian approaches.^39^ The Lagrangian approach defines the reference state as the system without adsorption, maintaining fixed thermodynamic conditions to establish a stable baseline for evaluating adsorption effects. Under this framework, structural properties, such as the accessible volume *V*_0_ before adsorption remain constant across different pressures. In contrast, the Eulerian approach compares the system with and without adsorption while maintaining identical mechanical conditions, facilitating a direct assessment of adsorption-induced phenomena. Consequently, structural properties before adsorption becomes pressure-dependent. These complementary approaches provide distinct yet synergistic perspectives, facilitating a comprehensive understanding of adsorption processes in nanoporous materials. This study created three models to understand better the adsorption and diffusion of H_2_, CH_4_, and CH_4_/H_2_ mixtures in type II-D kerogen matrix, incorporating both the Eulerian and Lagrangian approaches while accounting for kerogen swelling and deformation.

## Simulation Details

All molecular simulations were conducted using the LAMMPS software.^40^ The type II-D kerogen unit developed by Ungerer et al.^41^ was used to construct the kerogen matrix, representing overmature kerogen in the dry gas window, typical of reserves like Barnett shale. We constructed three kerogen matrix models with varying pore sizes by incorporating dummy particles with diameters of 0 nm (Model 1), 1 nm (Model 2), and 2 nm (Model 3) into the matrices. The procedure for constructing the kerogen matrices can be found in the Supporting Information (SI). Recognizing that the deformability and swelling of kerogen can significantly impact adsorption mechanisms, we investigated the adsorption of H_2_ and CH_4_ gases using both rigid and flexible kerogen models under typical shale reservoir conditions (363.15 K and pressure up to 50 MPa). For the flexible kerogen simulations, we utilized the osmotic ensemble.^25,42^ Specifically, every 1000 time steps under the NPT ensemble with a time step of 1 fs, we performed a GCMC cycle consisting of 500 insertion and deletion attempts to simulate gas adsorption. Also, the kerogen matrix was treated as static in the rigid state, with all atomic velocities constrained to zero.^43^ Similar to our previous works,^28,44^ we determined the fugacity-pressure relationship via additional GCMC and NPT ensemble MD simulations, comparing results under bulk conditions at the same density. The chemical potential values for the gases used in the GCMC simulations are listed in Table S2. These values were validated through GCMC simulations by comparing them with data from the NIST database^45^ and the Peng–Robinson equation of state^46^ (PR-EOS), as shown in Figure S1. A Nosé–Hoover thermostat utilizing a time constant of 100 fs maintains the temperature, while an anisotropic Nosé–Hoover barostat with a time constant of 1000 fs independently regulates the three diagonal components of the stress tensor, ensuring that the pressure within the system remains perfectly isotropic. GCMC simulations used the Metropolis algorithm, while MD simulations applied the velocity Verlet algorithm to solve Newton’s equations.^47^ The total simulation time was 15 ns, with the last 5 ns dedicated to data analysis. The simulation input files are available in SI.

The CVFF force field^48^ was used for kerogen. CH_4_ molecules were modeled using the TraPPE-UA force field,^49^ while H_2_ molecules were represented using the single-site Buch model,^50^ which can reproduce the bulk thermodynamic properties of H_2_ up to high pressures.^51^ Lorentz–Berthelot mixing rules were applied to determine the Lennard-Jones (LJ) parameters for unlike interactions. A cutoff radius of 1.4 nm was employed for nonbonded interactions without tail corrections.^52,53^ Long-range electrostatic interactions were computed using the PPPM method, achieving an accuracy of 10^–4^. Periodic boundary conditions were applied in all three spatial dimensions.

Self-diffusion arises from the movement of individual molecules due to Brownian motion. After achieving equilibrium through GCMC/MD simulations, MD simulations were performed in the NVE ensemble to calculate self-diffusion coefficients,^27,42^ which is based on the Einstein relation. This method determines the self-diffusion coefficient by analyzing the slope of the mean-squared displacement (MSD) in the diffusive regime^54^
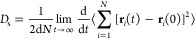
1where *D*_s_ is the self-diffusion coefficient, ⟨|**r**(*t*) – **r**(0)|^2^⟩ is MSD, *N* is the number of diffusing particles, *d* is the dimensionality of the system, *t* is time, and **r**(*t*) and **r**(0) represent the particle positions at time *t* and the initial time, respectively, and ⟨···⟩ denotes the ensemble average. Equation 1 is applicable only under the condition that the slope of the curve in the log(MSD)-log(*t*) plot equals unity. Unwrapped trajectories were recorded every 1 ps for 60 ns, and only the first half of the simulation points were considered time origins to ensure equal weight. Diffusion coefficients were obtained using the diffusion coefficient analysis tool available in VMD.^55^

## Results and Discussion

### Adsorption

**Figure 1:**
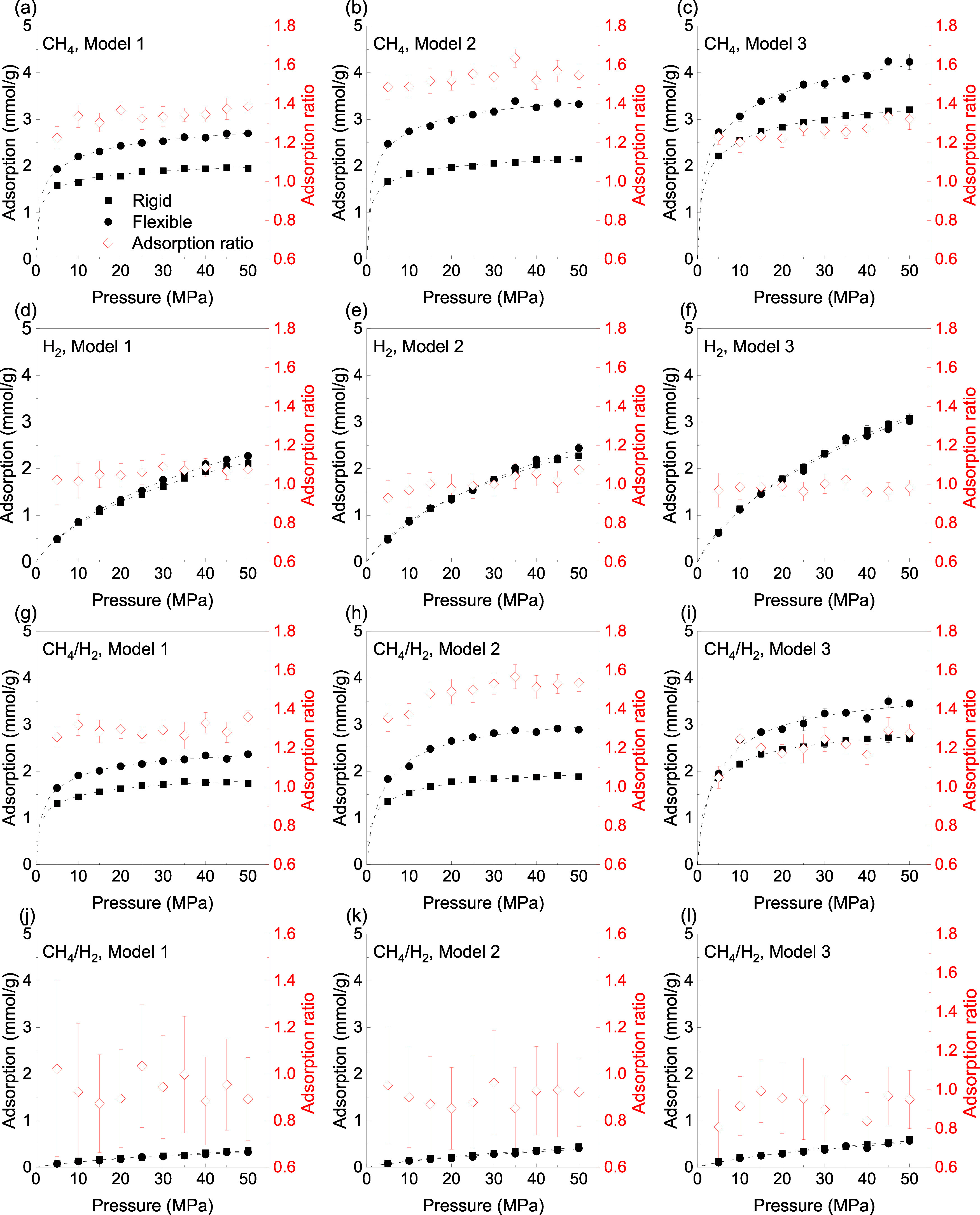
Absolute adsorption at *T* = 363.15 K using the Lagrangian approach: (a–c) CH_4_ adsorption; (d–f) H_2_ adsorption; (g–i) CH_4_ adsorption in a CH_4_/H_2_ mixture; (j–l) H_2_ adsorption in a CH_4_/H_2_ mixture, for different models. The adsorption ratio is the amount of gas adsorbed in flexible kerogen relative to rigid kerogen. The dashed lines indicate the fitted curves based on the Tóth equation.

Figure 1 presents the adsorption behavior of CH_4_, H_2_, and CH_4_/H_2_ mixture (1:1) across different models in rigid and flexible states. The adsorption of CH_4_ significantly alters the kerogen pore structure, resulting in pore swelling and an increased adsorption capacity compared to rigid kerogen. This increase depends on the kerogen’s pore size distribution (PSD). Notably, the adsorption capacity of CH_4_ in flexible kerogen models is approximately 1.4, 1.6, and 1.3 times higher than that of rigid kerogen at 50 MPa for Models 1, 2, and 3, respectively. These findings align with other studies,^23,24,26^ demonstrating an increase in CH_4_ adsorption compared to the rigid kerogen. In contrast, the adsorption of H_2_ demonstrates negligible differences between rigid and flexible kerogen across all models. This can be attributed to the relatively small molecular size and weak interaction energy of H_2_ molecules with the kerogen matrix. Hydrogen’s low affinity for the kerogen structure results in minimal impact from the kerogen’s flexibility. Furthermore, hydrogen’s high diffusivity and low condensation tendency under the studied conditions likely reduce the influence of pore size variations, leading to similar adsorption behaviors in both rigid and flexible kerogen models. Therefore, the flexibility of kerogen has a more pronounced effect on CH_4_ adsorption than on H_2_, reflecting the different physical properties and adsorption mechanisms of these two gases. Figure 1g–i presents the CH_4_ adsorption in a CH_4_/H_2_ mixture, while Figure 1j–l illustrates the H_2_ adsorption in the same mixture. As observed, similar to the pure component states, the presence of CH_4_ induces swelling and kerogen deformation. The CH_4_ adsorption ratio in the flexible state is comparable to that in the rigid state, akin to the behavior observed in pure CH_4_. However, the CH_4_ adsorption in the mixture is less compared to pure CH_4_, likely due to the presence of H_2_. A similar trend is observed for H_2_ adsorption, where the results mirror those of pure H_2_, with CH_4_ leading to a reduction in H_2_ adsorption.

Also, Figure 1 presents data fitted using the Tóth isotherm model.^56^ The following equation describes the model
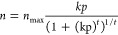
2where *n* represents the loading at various equilibrium pressures, *n*_max_ is the theoretical maximum adsorption capacity, *p* is pressure, and *k* is the Tóth equilibrium constant, reflecting the affinity between the adsorbate and adsorbent—higher *k* values indicate stronger binding. The parameter *t* characterizes the heterogeneity of adsorption sites, which *t* < 1 means greater heterogeneity while *t* ≈ 1 homogeneity (consistent with Langmuir adsorption). Data sets are well-fitted with the Tóth isotherms in the entire pressure range. The fitted parameters for each sample are provided in Table S3. In analyzing the kerogen structure, the *t* values for CH_4_ are found to be 0.40 and 0.34 in rigid and flexible states, respectively, while H_2_ values are 0.53 and 0.45. These values reveal the structural heterogeneity within kerogen and demonstrate that incorporating kerogen flexibility leads to lower *t* values. This reduction infers that kerogen’s flexible state enhances molecular accessibility and alters the binding site distribution, influencing adsorption behavior.

**Figure 2:**
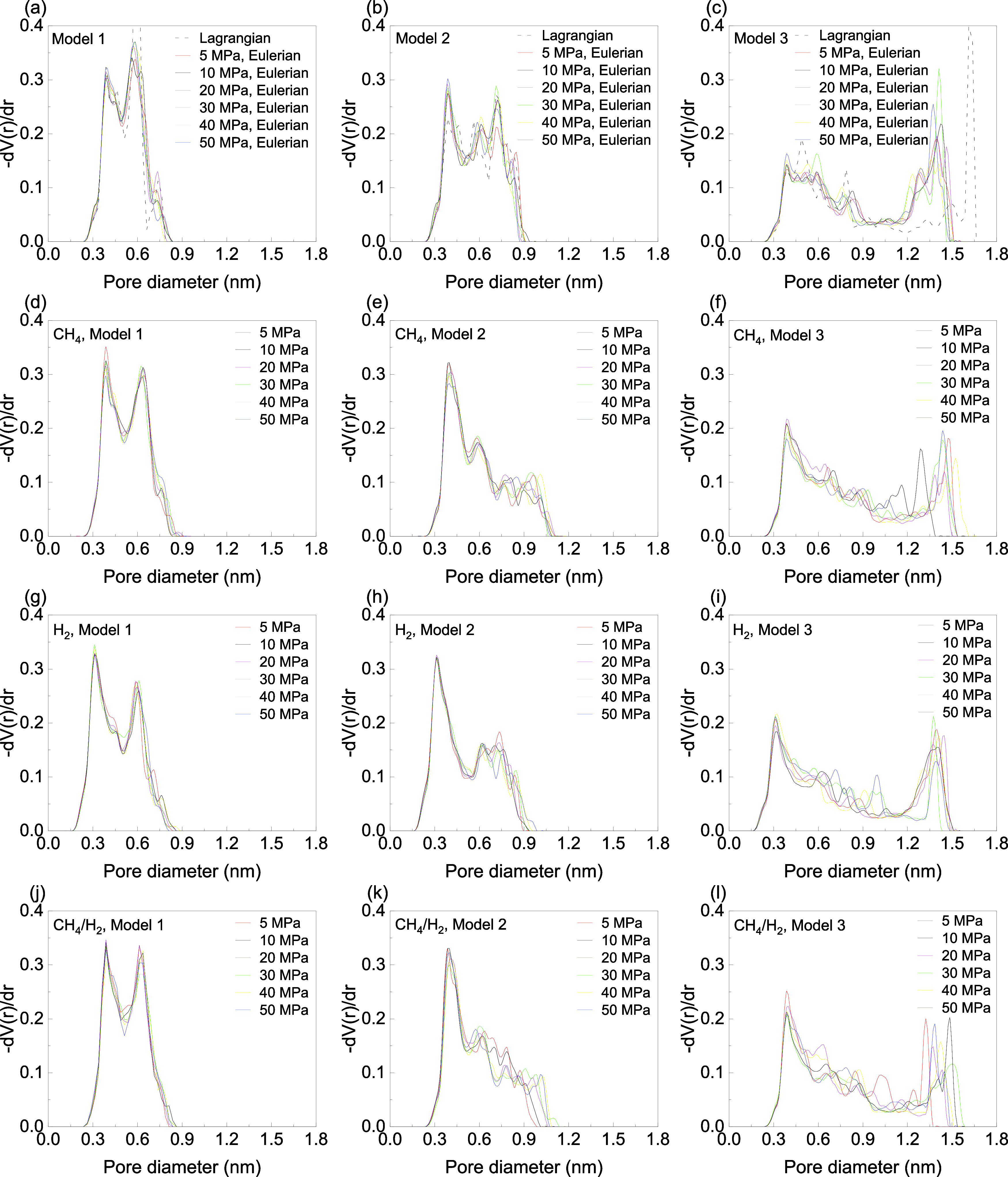
Pore size distribution (a) Model 1, (b) Model 2, (c) Model 3 before adsorption for both the Eulerian and Lagrangian approaches; (d) Model 1, (e) Model 2, and (f) Model 3 after CH_4_ adsorption; (g) Model 1, (h) Model 2, (i) Model 3 after H_2_ adsorption; (j) Model 1, (k) Model 2, Model 3 (l) after CH_4_/H_2_ mixture adsorption at *T* = 363.15 K. Error bars are not displayed to enhance the visibility of the figures.

**Figure 3:**
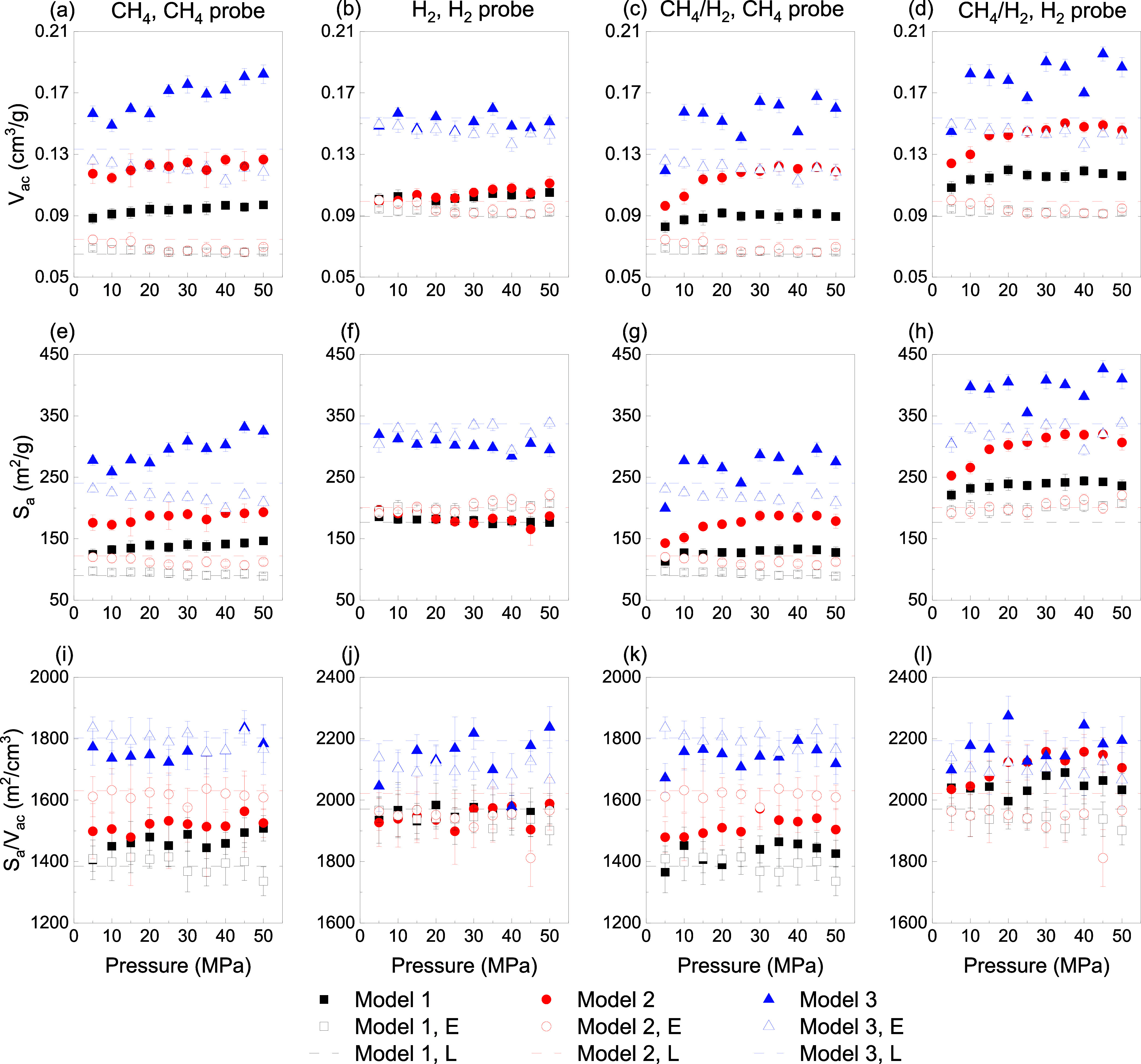
Evolution of the accessible volume: (a) CH_4_, (b) H_2_, (c, d) CH_4_/H_2_ mixture; specific surface area: (e) CH_4_, (f) H_2_, (g, h) CH_4_/H_2_ mixture; specific surface area/accessible volume: (i) CH_4_, (j) H_2_, (k, l) CH_4_/H_2_ mixture as a function of pressure, with a comparison between the Lagrangian (L) and Eulerian (E) approaches.

The further analysis explores the impact of CH_4_ and H_2_ adsorption on the pore structure, including changes in PSD and geometric properties as illustrated in Figures 2 and 3, respectively. The PSDs were described as the statistical distribution of the diameters of the largest spheres that could fit within a pore at specific points. These were determined using PoreBlazer v.4 software,^57^ which relies on the van der Waals (vdW) volume of kerogen atoms for its calculations. Figure 2a-c shows the PSD before adsorption based on the Eulerian and Lagrangian approaches. The Lagrangian approach is conducted at a lower pressure (0.1 MPa) than the Eulerian approach, resulting in larger pore sizes, especially in Model 3. In contrast, under the Eulerian approach, an increase in pressure reduces both the accessible volume and the PSD. Therefore, the choice of approach significantly impacts the amount and trend of volumetric strain, which will be discussed later. For CH_4_ adsorption (Figure 2d–f), Model 2 exhibits an increase in accessible volume at low pressures for larger pores, accompanied by a slight shift toward larger pore sizes. This trend stabilizes at higher pressures as pore size restrictions limit further gas insertion. However, in Models 1 and 3, the most significant change is the formation of additional pores within the 0.31–0.48 nm size range, leading to considerable swelling. As gas pressure increases, kerogen swelling allows gas to access previously inaccessible areas, a behavior supported by the PSD results for CH_4_ at different pressures. Additionally, Model 2 exhibits the largest increase in pore size within the PSD, which correlates with the higher CH_4_ adsorption observed in this model compared to the rigid case, as shown in Figure 1b. These results imply that gas adsorption not only increases the number of pores at a specific pore size but also contributes to the formation of larger pores through swelling. Specifically, during CH_4_ adsorption, the pores in the kerogen become significantly larger than during H_2_ adsorption under the same pressure conditions. This suggests that CH_4_ adsorption induces larger structural changes in the kerogen matrix, enhancing gas accessibility and swelling.

Figure 3 depicts the evolution of specific surface area *S*_a_ and accessible volume *V*_ac_ with applied pressure. In CH_4_ adsorption, a methane-sized probe was used to calculate both *S*_a_ and *V*_ac_, while a hydrogen probe was used for H_2_. In the CH_4_/H_2_ mixture, both CH_4_ and H_2_ probe sizes were used. In Model 1, which has the smallest pore size, both the Lagrangian and Eulerian approaches yield identical quantities of pore volume. This consistency is due to the limited dimensions of the pores, which restrict significant changes in pore volume under increased pressure within the Eulerian approach. However, as pore size increases, the discrepancy between these two approaches becomes more pronounced. Larger pores experience greater compression under pressure, resulting in a noticeable reduction in volume. A similar trend is observed in the *S*_a_. Model 2 reveals the most significant change in volume among the models for CH_4_ adsorption, with an overall increase. When using the Lagrangian approach, the volume increase at 50 MPa is observed to be 1.49, 1.70, and 1.37 times for Models 1, 2, and 3, respectively. This denotes a nonlinear response to pressure that depends on pore size. Similarly, the Eulerian approach causes an increase in volume by 1.46, 1.82, and 1.54 times, respectively. This highlights a slightly higher sensitivity to pressure in Model 2 compared to the Lagrangian method which suggests that the Eulerian approach may better capture pore volume changes in larger-pore systems under high-pressure conditions.

In contrast, H_2_ behavior deviates significantly from that observed for CH_4_. Considering H_2_, the Lagrangian approach indicates the greatest volume change in Model 1, while the Eulerian approach reveals the highest volume change in Model 2. Specifically, the volume changes in the Eulerian approach at 50 MPa are 1.13, 1.17, and 1.06 times for Models 1, 2, and 3, respectively, implying that larger pores undergo reduced compression. In the Lagrangian approach, the corresponding values at 50 MPa are 1.17, 1.12, and 0.98 times. Notably, the volume changes for H_2_, when considering the error bars, remain relatively similar across the models, with negligible swelling effects. This means that, in H_2_ case, the pore structure exhibits limited sensitivity to pressure variations, leading to minimal volumetric expansion or compression compared to CH_4_. The observed difference between CH_4_ and H_2_ underscores the importance of gas-specific interactions with the pore structure in influencing the compression and adsorption behavior. These findings emphasize the importance of considering both gas type and pore size when modeling adsorption processes. The differing trends observed between the Lagrangian and Eulerian approaches further highlight the necessity of selecting an appropriate computational framework based on the physical properties of the gas and the pore structure of the material.

In the adsorption of CH_4_ in the kerogen matrix, it would be expected that swelling would lead to an increase in pore size, thereby reducing the surface-to-volume ratio *S*_a_/*V*_ac_ of the pores. However, as shown in Figure 3i, this trend is not observed in the models, and *S*_a_/*V*_ac_ remains almost constant. This is also confirmed by examining the evolution of the PSD with pressure (Figure 2d–f), which reveals minimal variation within the error bars. Therefore, swelling-induced changes in pore structure are considered complex and may involve localized deformation or restructuring of the kerogen matrix, depending on the pressure and adsorption conditions. A similar trend is found in H_2_ adsorption. Due to H_2_ smaller molecular size than CH_4_, *S*_a_/*V*_ac_ is higher for H_2_. However, since the swelling induced by H_2_ adsorption is negligible, the volume reduction remains constant and does not exhibit significant variation, remaining within the simulation error margins. Additionally, as expected, the trends in *S*_a_ and *V*_ac_ in the CH_4_/H_2_ mixture closely follow those observed for CH_4_, due to the greater influence of methane on swelling and deformation.

**Figure 4:**
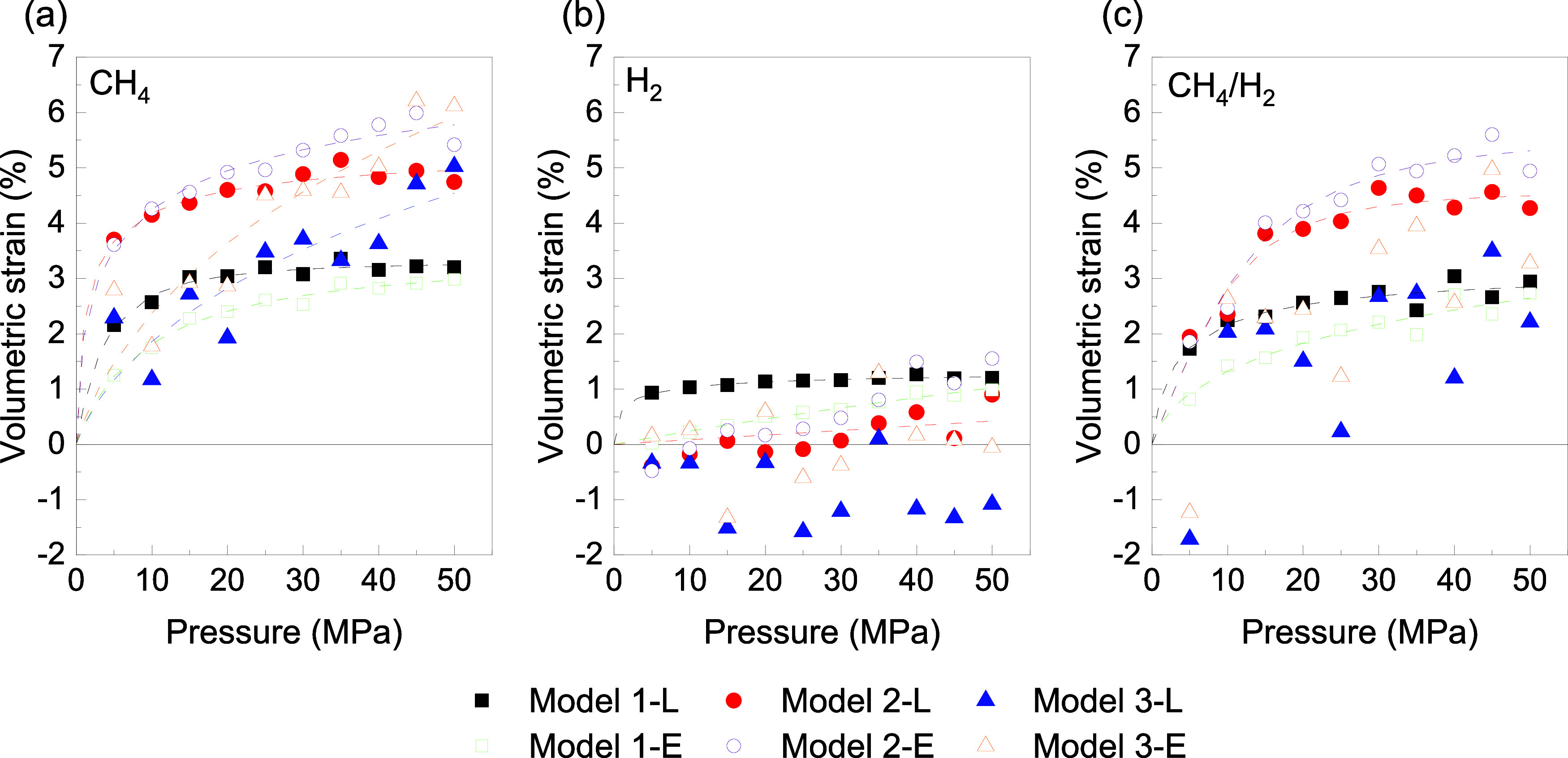
Volumetric strain-pressure in (a) CH_4_, (b) H_2_, and (c) CH_4_/H_2_ mixture for the Lagrangian (L) and Eulerian (E) approaches. Dashed line: Tóth isotherm fit.

**Figure 5:**
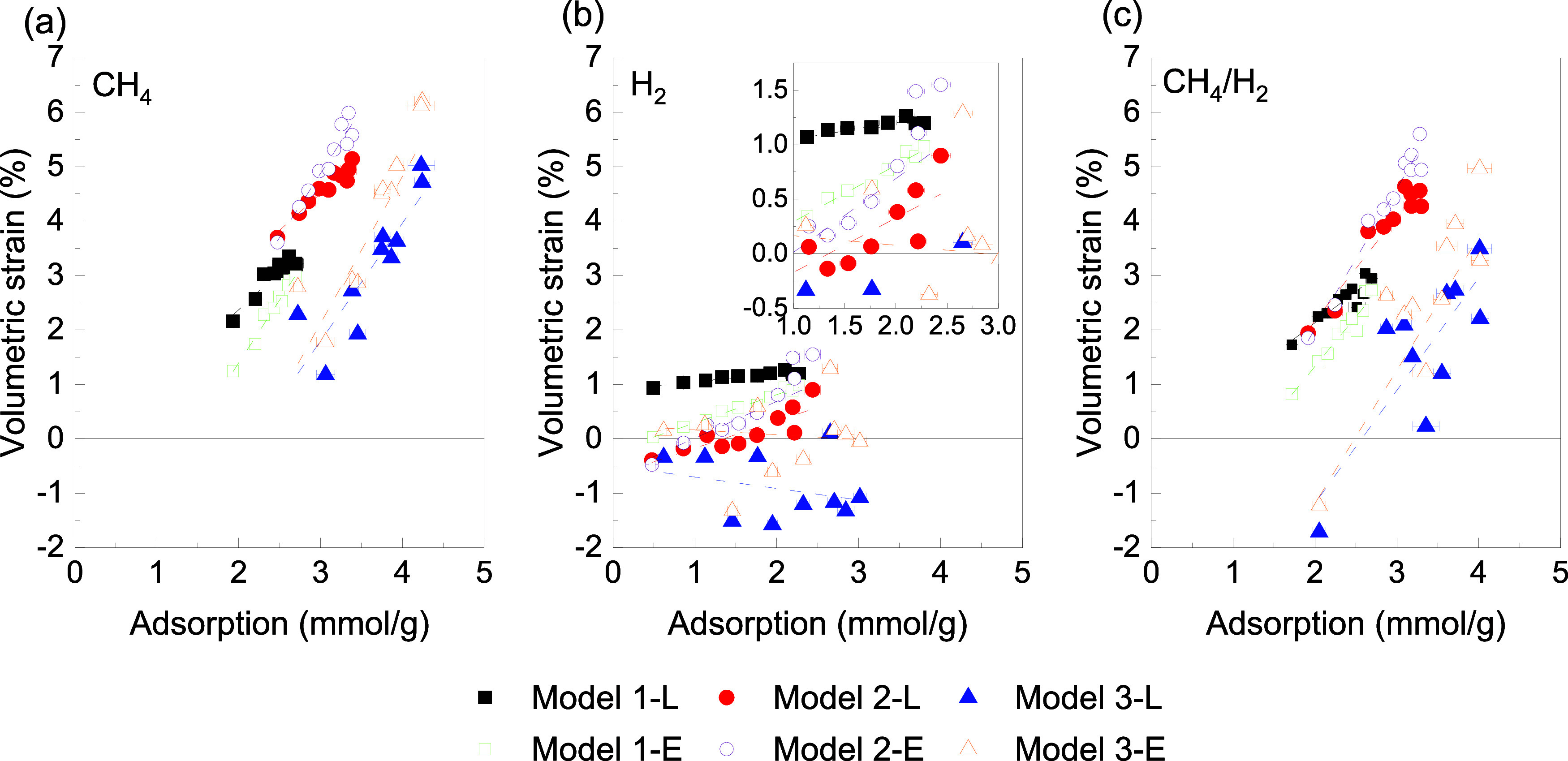
Volumetric strain-adsorption in (a) CH_4_, (b) H_2_, and (c) CH_4_/H_2_ mixture for the Lagrangian (L) and Eulerian (E) approaches. Dashed line: linear fit.

To further examine the significance of the Eulerian and Lagrangian approaches, volumetric strain as a function of pressure is presented in Figure 4, while volumetric strain as a function of loading is shown in Figure 5. The volumetric strain of the matrix induced by adsorption is defined as , where *V* and *V*_0_ represent the average matrix volumes computed after adsorption from GCMC/MD simulations at pressure *P* and before adsorption with Eulerian and Lagrangian approaches, respectively. For CH_4_, a clear increase in ε_*v*_ is observed with increasing pressure, indicating that adsorption leads to a noticeable expansion of the kerogen matrix. Using the Lagrangian approach, the ε_*v*_ at 50 MPa is between 3.25 and 4.54% for these three different models, while in the Eulerian approach, it ranges from 2.97 to 5.92%. These results are consistent with the studies by Yang et al.^32^ and Wu et al.,^31^ which reported similar trends in ε_v_ under similar conditions.

This increase in ε_*v*_ is attributed to the significant swelling of the kerogen matrix as CH_4_ molecules are adsorbed, with the Lagrangian approach typically predicting a larger strain since a lower pressure environment is used in this method. In contrast, the Eulerian approach, which simulates adsorption under higher pressures, results in a relatively smaller but still notable strain increase. The difference between the two approaches delineates the impact of pressure on matrix deformation, with the Eulerian approach showing a higher strain at higher pressures. Additionally, the discrepancy in strain values across different models suggests that the matrix’s initial structural properties, such as PSD and flexibility, play a key role in determining the extent of volumetric expansion.

Unlike CH_4_, in H_2_, as the pore size increases from Model 1 to Model 3, the amount of swelling decreases, and contraction occurs instead of swelling. This behavior can be attributed to the small molecular size of H_2_ and its weak interaction with the kerogen matrix. These factors make the effect of pressure more significant, causing the H_2_ molecules to be less likely to induce pore expansion. Furthermore, the low adsorption affinity of H_2_ for the kerogen structure may reduce pore volume, leading to the observed contraction. Consequently, the interplay between hydrogen’s small molecular size, weak adsorption, and pressure conditions leads to a counterintuitive contraction rather than the expected swelling.

However, in the CH_4_/H_2_ mixture, as mentioned earlier, the effects of CH_4_ are greater than those of H_2_, which leads to swelling of the matrix. Contraction occurs at low pressures, particularly in Model 3, instead of swelling. This behavior is typically observed at lower pressures, where the system tends to shrink as cavities deform to better accommodate the adsorbed gas molecules. As the pressure increases, the system subsequently swells to accommodate more gas molecules. For instance, under the Lagrangian approach, the volumetric strain at a pressure of 50 MPa ranges from 2.55 to 4.50%, while for the Eulerian approach, it varies from 2.64 to 5.31%. An interesting observation is that the trend of ε_*v*_ can vary depending on the pore size, type of gas, and gas composition. Also, in Figure 4, the Tóth model fitting curves on the data with good fit are shown, with ε_v_ replaced by *n* in eq 2. As observed, the Tóth model fails to adequately fit the data exhibiting negative ε_v_, especially for H_2_, and these data are excluded from the figure. These factors collectively influence the matrix deformation, suggesting that different gas types and their interactions with the kerogen matrix can lead to distinct strain behaviors. These findings underscore the importance of choosing the appropriate approach for accurately predicting adsorption-induced matrix deformation, as both pressure conditions and model parameters significantly influence the observed ε_v_.

As shown in Figure 5a, CH_4_ loading and ε_v_ exhibit a strong linear correlation, consistent with other studies^23,29,30,33,37,38^ on kerogen and experiments^58^ on shale samples. This relationship suggests that the increased porosity resulting from swelling can accommodate more CH_4_ molecules. Our models, with their distinct initial pore structures, exhibit varying deformation responses to CH_4_ adsorption, based on various slopes of the linear fitting. Notably, Model 3, which has the largest initial pore volume, exhibits the smallest swelling at the same CH_4_ loading, while Model 2, with a moderate initial pore volume, shows the largest ε_v_, following the order: Model 2E > Model 2L > Model 1E > Model 1L > Model 3E > Model 3L. This implies that higher local pressures and stresses in smaller pores do not always result in larger deformation which confirms the critical influence of initial pore structure on adsorption-induced deformation in porous media. However, as shown in Figure 4b, Figure 5b demonstrates that ε_v_-adsorption in H_2_ does not follow a uniform trend, with kerogen either swelling or contracting depending on pore size. This originates from the distinct behavior of H_2_ when interacting with the same structure but varying geometric properties, which warrants further investigation to understand hydrogen’s behavior. Also, the divergence between Lagrangian and Eulerian approaches becomes particularly pronounced at high pressures for H_2_, with trends strongly modulated by pore size distribution (Model 3). In contrast, the linear behavior observed for the CH_4_/H_2_ system (Figure 5c) is related to the relatively stronger influence of CH_4_ compared to H_2_ in terms of adsorption within the kerogen matrix. As previously discussed, CH_4_, being larger and more nonpolar, exhibits a higher affinity for the kerogen surface, leading to more significant adsorption. This results in a more predictable, linear relationship in the adsorption isotherms for the CH_4_/H_2_ mixture. The adsorption of H_2_, being smaller and more diffusible, is less affected by the kerogen structure, which explains the less pronounced deviations from the linear trend for the CH_4_/H_2_ mixture. This reveals the molecular interactions occurring at the kerogen surface and the importance of understanding the competitive adsorption behaviors of different gases in porous media for optimizing gas storage and extraction processes.

Adsorption selectivity, a key factor in determining the relative adsorption priority in binary mixtures, is defined as the ratio of the mole fractions of two species in the adsorbed phase to their ratio in the bulk phase.^18^ This can be calculated using the following equation
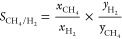
3where *x*_CH_4__ and *x*_H_2__ are the mole fractions of CH_4_ and H_2_ in the adsorbed phase, and *y*_CH_4__ and *y*_H_2__ represent their respective mole fractions in the bulk phase.

**Figure 6:**
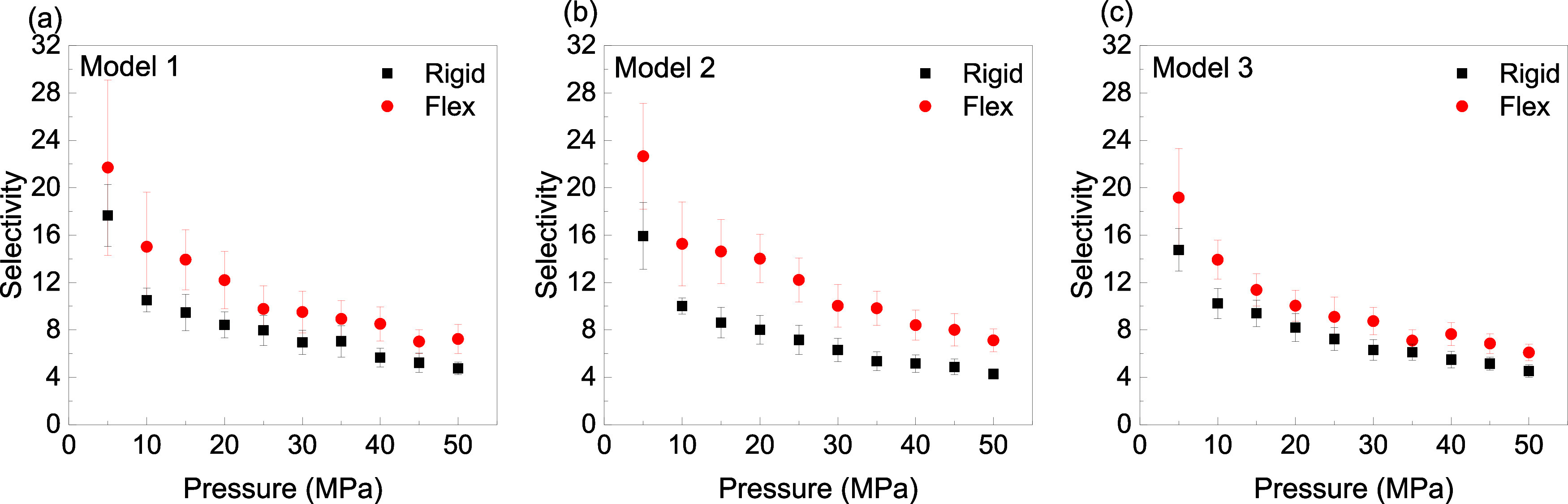
Selectivity of CH_4_/H_2_ (1:1) at *T* = 363.15 K using the Lagrangian approach: (a) Model 1, (b) Model 2, and (c) Model 3.

In Figure 6, the selectivity of CH_4_/H_2_ (1:1) for both rigid and flexible kerogen is presented. As observed, the selectivity is consistently greater than 1 across all pressures, demonstrating a stronger kerogen affinity for CH_4_ than H_2_. This denotes that CH_4_ is preferentially adsorbed over H_2_ in the kerogen structures. However, with increasing pressure, the selectivity decreases, which shows at higher pressures, the adsorption of CH_4_ becomes less favorable relative to H_2_. This is not unexpected in adsorption processes, as increased pressure often leads to saturation of adsorption sites, reducing the distinction in adsorption behavior between the two species. Moreover, it is noteworthy that flexible kerogen consistently exhibits higher selectivity than rigid kerogen. This enhanced selectivity in flexible kerogen can arise from the increased conformational adaptability of the matrix, allowing for more favorable interactions with CH_4_ molecules. The flexibility likely increases the available microporous volume or alters the pore geometry, enhancing the capacity for CH_4_ adsorption relative to H_2_. This phenomenon reveals how kerogen’s structural properties would control its adsorption behavior, particularly in heterogeneous materials.

### Self-Diffusion Coefficient

In this section, we investigate the self-diffusion coefficient in kerogen matrices. As summarized in Table 1, the CH_4_ self-diffusion coefficient within kerogen matrices, as developed by Ungerer et al.,^41^ exhibits a major dependence on the methodology employed for constructing the kerogen matrix and the number of kerogen units considered, leading to variations in porosity. Also, because of the small size of the constituent molecules and limited cross-linking, these models primarily display ultramicroporosity.^9^ Expanding the porosity range necessitates specific methods during bulk model generation, such as incorporating fluid molecules^59,60^ or dummy particles^26,61^ of varying sizes. Notably, the self-diffusion coefficient of CH_4_ spans in a range from 10^–12^ to 10^–9^ m^2^/s in Ungerer kerogen (Table 1). Ungerer kerogen models, based on packing small identical molecules, accurately represent chemistry and host–guest interactions but may not capture poromechanics due to their limited component size.^9^ Models like those from hybrid reverse Monte Carlo (HRMC)^62^ or replica exchange molecular dynamics (REMD)^63^ offer better alternatives, though they might be less realistic from a chemistry perspective.

**Table 1:** Self-Diffusion Coefficients of CH_4_ in the Kerogen Matrix of Ungerer et al.

refs	*D*_s_ (10^–9^ m^2^/s)	kerogen info	*T* (K)	*P* (MPa)
Tesson and Firoozabadi^26^	6.1–7.1	12 II-A^a^	333.15	14
Vasileiadis et al.^61^	0.0016–3.5567	50 II-D^a^	298.15	0.1
Perez and Devegowda^59^	0.6677	80 II–C^b^	355	30
Afagwu et al.^60^	1.44–2.90	5 II-D^b^	350	2.07–41.34
Pathak et al.^64^	2	15 II–C	400	30
Sui et al.^65^	1.4–3.10	4 II-A	298–380	1–30
Huang et al.^35^	0.031–0.059	7 III-A	318	
Ho et al.^66^	0.03–4.4	24 II-D	338	2–30
Gong et al.^34^	0.0131	10 II-A	298	10
	0.0355	11 II–B		
	0.0758	11 II–C		
	0.0179	16 II-D		
Yuan et al.^67,68^	0.037–0.046	10 I-A	338	5–25
	0.172–0.186	10 II-A		
	0.176–0.187	10 II–B		
	0.177–0.190	10 II–C		
	0.180–0.185	10 II-D		
	0.087–0.103	10 III-A		
Yu et al.^69^	0.198–0.215	50 I-A	365	27.5
	0.003–0.005	50 II-A		
	0.008–0.014	50 II–B		
	0.009–0.018	50 II–C		
	0.014–0.047	50 II-D		
	0.010–0.029	50 III-A		
Zhang et al.^70^	0.115	50 I-A	365	27.5
	0.008	50 II-A		
	0.030	50 II–B		
	0.025	50 II–C		
	0.021	50 II-D		

a Construct the kerogen matrix using dummy particles.

b Construct the kerogen matrix using specific molecules.

We evaluated the diffusion coefficients of CH_4_ and H_2_ in flexible-state Model 3, which has a larger pore volume that facilitates diffusion under a constant pressure of 5 MPa and a temperature of 363.15 K. The MSD as a function of time as shown in Figure S2. The self-diffusion coefficient for CH_4_ was determined to be 0.30 × 10^–9^ m^2^/s, while this value for H_2_ was significantly higher at 6.87 × 10^–9^ m^2^/s. The results align with Ho et al.,^19^ where they found H_2_ diffusion is about 1 order of magnitude higher than CH_4_ in kerogen. Additionally, to investigate the anisotropic behavior of kerogen, diffusivity was calculated along the *x*, *y*, and *z* axes for Model 3. Considering CH_4_, the diffusivity values in the *x* and *z* directions were 0.16 × 10^–9^ and 0.73 × 10^–9^ m^2^/s, respectively, while diffusivity in the *y* direction was negligible. In contrast, H_2_ exhibited diffusivity values of 10.72 × 10^–9^, 2.53 × 10^–9^, and 7.34 × 10^–9^ m^2^/s in the *x*, *y*, and *z* directions, respectively. To investigate the impact of pressure on the diffusion rate, the self-diffusion coefficient of H_2_ was calculated at a pressure of 50 MPa using Model 3 kerogen in a flexible state. As expected, the self-diffusion coefficients (0.45 × 10^–9^ m^2^/s) decreased with increasing pressure. This trend aligns with gas diffusion in nanoporous materials, where elevated pressures result in a diminished space for molecular movement, thereby restricting the mobility of gas molecules during diffusion. Moreover, the three-dimensional diffusivity for H_2_ in Model 1 at a pressure of 5 MPa is 0.17 × 10^–9^ m^2^/s, which proves that diffusivity is highly controlled by the structural configuration of the kerogen matrix. This being said, how the kerogen model is constructed would significantly influence its pore architecture, resulting in considerable variations in diffusivity. This underscores the critical role of matrix preparation techniques in controlling H_2_ diffusion behavior within microporous kerogen, thereby impacting the overall storage calculations. These results demonstrate that H_2_ diffusion is significantly higher than CH_4_ diffusion across all measured directions, proving the directional dependence of gas diffusion within the kerogen matrix. The higher diffusivity of H_2_ can be attributed to its smaller molecular size and weaker interactions with the kerogen structure, facilitating more efficient transport through the microporous network. Understanding these anisotropic diffusion behaviors is critical for optimizing H_2_ storage strategies in microporous, organic-rich geological formations.

## Conclusions

Our simulations of H_2_, CH_4_, and their mixtures at a temperature of 363.15 K and pressure up to 50 MPa within kerogen matrices with varying pore size distributions emphasize the critical role of adsorption-induced swelling in these systems. At elevated pressures, CH_4_ adsorption increases by approximately 40% than the rigid-state matrix, demonstrating the significant impact of matrix flexibility. In contrast, H_2_ adsorption is highly sensitive to pore size distribution, leading to either matrix swelling or contraction. A comparative analysis of volumetric strain using the Eulerian and Lagrangian approaches consistently shows higher strain estimates with the Eulerian method. However, the divergence between these two methods becomes particularly pronounced at high pressures for H_2_, with trends strongly modulated by pore size distribution. The Tóth isothermal model effectively describes the adsorption behaviors of H_2_ and CH_4_ in flexible and rigid kerogen matrices, and CH_4_ volumetric strain isotherms. However, when contraction occurs, the Tóth model fails to accurately describe the volumetric strain isotherms for H_2_ and CH_4_/H_2_ mixtures. Additionally, our simulations indicate that H_2_ diffusion in porous kerogen is 1 order of magnitude faster than that of CH_4_. The diffusion characteristics of kerogen matrices, particularly those based on the Ungerer model,^41^ are highly sensitive to the methodology used for matrix construction and the number of kerogen units modeled, underscoring the importance of these parameters in obtaining accurate simulation results.

## Supporting Information Available

Details on the construction of the kerogen matrix, chemical potentials used in GCMC simulations, Tóth model parameters, and mean-squared displacement of CH_4_ and H_2_ (PDF)
LAMMPS input files (ZIP)
