# Supplementary material for: Adsorption-Induced Deformation in Microporous Kerogen by Hydrogen and Methane: Implications for Underground Hydrogen Storage
Source: Langmuir. 2025 Mar 3;41(9):6364–75. doi: 10.1021/acs.langmuir.5c00197 (PMC11912540; doi:10.1021/acs.langmuir.5c00197)
Supplement: Supplementary file 1 — la5c00197_si_001.pdf [file la5c00197_si_001.pdf]

Supporting Information: Adsorption-Induced Deformation in Microporous Kerogen by Hydrogen and Methane: Implications for Underground Hydrogen Storage

Saeed Babaei,† Benoit Coasne,*,‡,¶ and Mehdi Ostadhassan*,§

†*Civil Engineering Faculty, K. N. Toosi University of Technology, Tehran 1969764499, Iran*

‡*University Grenoble Alpes, CNRS, LIPhy, 38000 Grenoble, France*

¶*Institut Laue Langevin, F-38042 Grenoble, France*

§*Institute of Geosciences, Marine and Land Geomechanics and Geotectonics, Christian-Albrechts Universität, Kiel 24118, Germany*

E-mail: benoit.coasne@univ-grenoble-alpes.fr; mehdi.ostadhassan@ifg.uni-kiel.de

Content

Kerogen Matrices Generation 2

Force Field Validation 3

Mean-Square Displacement of CH_4 and H_2 3

Chemical Potential of Gases 4

Fitting Parameters for the Toth Model 4

References 5

Kerogen Matrices Generation

We constructed three kerogen matrix models with varying pore sizes by incorporating dummy particles (DPs) with diameters of 0 nm (Model 1), 1 nm (Model 2), and 2 nm (Model 3) into the matrices. The type II-D kerogen unit ($C_{175}H_{102}O_9N_4S_2$) developed by Ungerer et al.[1] was used to construct the kerogen matrix, representing overmature kerogen in the dry gas window, typical of reserves like Barnett shale. First, 24 kerogen units were arranged without overlap within a cubic simulation cell of dimensions $10 \times 10 \times 10$ nm^3 to build the kerogen matrix. The kerogen matrix is constructed by following the procedures in Table S1. Furthermore, for Models 2 and 3, following stage 7, the DPs were removed, and an additional run was conducted under the NPT ensemble at 300 K and 0.1 MPa for 1 ns to relax the system further. Models 1, 2, and 3 demonstrated largest cavity diameters (LCD) of 0.77 nm, 0.86 nm, and 1.64 nm, respectively. Notably, the LCDs of Models 2 and 3 were smaller than the diameter of the DPs. This discrepancy can be attributed to the relaxation of the matrix following the removal of the DPs. The resulting mean densities for the kerogen matrices in Models 1, 2, and 3 were 1.31, 1.29, and 1.22 g/cm^3, respectively. These values are within the range of experimental (1.3–1.4 g/cm^3)[2] and simulation (1.2–1.3 g/cm^3)[3] results for type II-D kerogen density.

Table S1: Molecular dynamics relaxation procedure to create the kerogen matrix.

Stage	Ensemble	Temperature (K)	Pressure (MPa)	Time (ns)
1	NVT	900	-	0.3
2	NPT	900	20	0.3
3	NPT	$900 \rightarrow 700$	20	0.3
4	NPT	$700 \rightarrow 500$	20	0.3
5	NPT	$500 \rightarrow 300$	20	0.3
6	NPT	300	$20 \rightarrow 0.1$	0.3
7	NPT	300	0.1	1

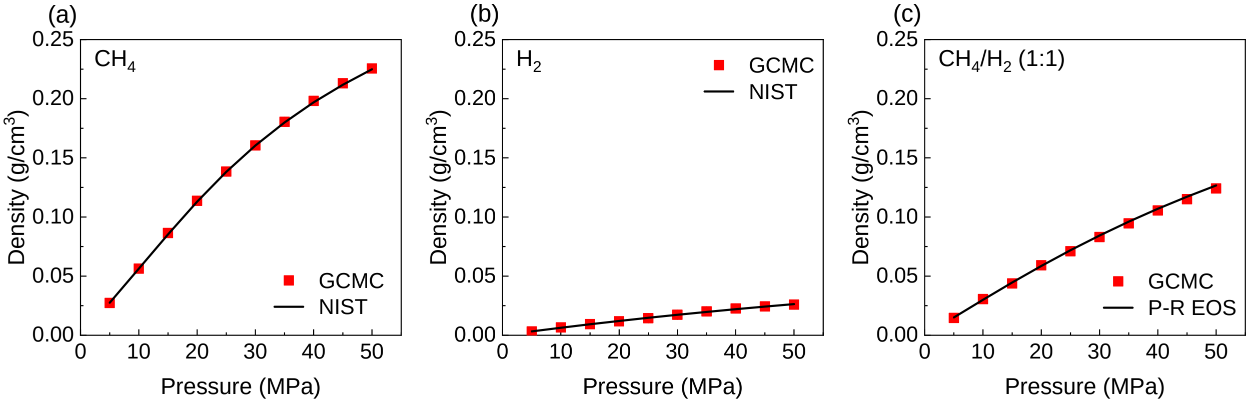

Figure S1: Comparison of bulk density of (a) CH_4 (b) H_2 and (c) CH_4/H_2 mixture obtained from GCMC simulation with NIST and Peng-Robinson equation of state at 363.15 K.

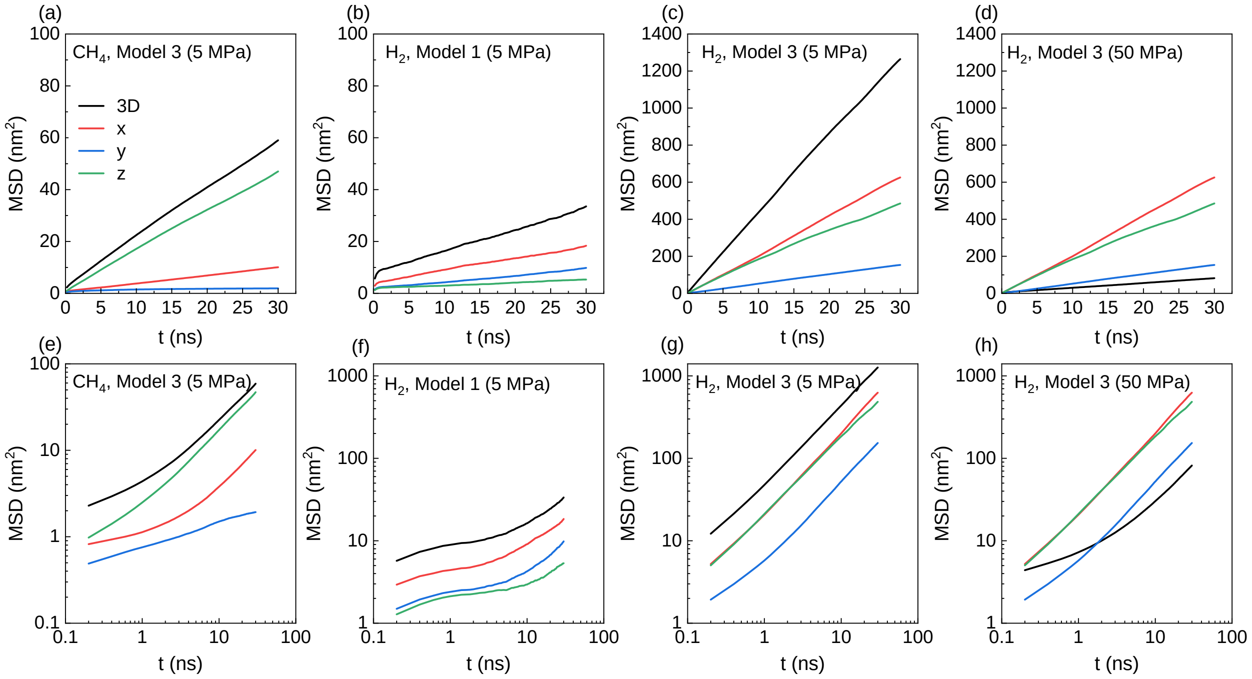

Figure S2: Mean-square displacement (MSD) of (a, e) CH_4 in flexible Model 3 at 5 MPa, (b, f) H_2 in flexible Model 1 at 5 MPa (c, g) H_2 in flexible Model 3 at 5 MPa, and (d, h) H_2 in flexible Model 3 at 50 MPa, presented on both linear and log-log scales.

Table S2: Chemical potentials (kJ/mol) used for GCMC simulations at T=363.15 K.

Pressure (MPa)	CH_4	H_2	CH_4 (CH_4/H_2)	H_2 (CH_4/H_2)
5	-8.19	-5.92	-8.70	-6.46
10	-7.72	-5.37	-8.17	-5.87
15	-7.43	-5.08	-7.91	-5.58
20	-7.24	-4.88	-7.68	-5.34
25	-7.09	-4.70	-7.53	-5.17
30	-6.95	-4.53	-7.39	-5.01
35	-6.83	-4.37	-7.28	-4.88
40	-6.73	-4.26	-7.17	-4.76
45	-6.63	-4.17	-7.08	-4.66
50	-6.54	-4.11	-6.99	-4.55

Table S3: Fitted parameters of the Tóth model.

			n_{max} (mmol/g)	k (MPa^{-1})	t	R^2	RMSE
CH_4	Rigid	Model 1	2.366	16.694	0.387	0.956	0.027
		Model 2	2.575	9.736	0.411	0.980	0.021
		Model 3	4.169	3.919	0.409	0.996	0.018
	Flex	Model 1	3.789	16.021	0.323	0.995	0.016
		Model 2	4.179	4.594	0.426	0.955	0.060
		Model 3	7.547	10.483	0.275	0.969	0.082
H_2	Rigid	Model 1	7.426	0.021	0.541	0.998	0.022
		Model 2	11.638	0.017	0.449	0.999	0.021
		Model 3	10.684	0.017	0.608	0.998	0.034
	Flex	Model 1	10.318	0.016	0.503	0.998	0.026
		Model 2	16.746	0.010	0.451	0.996	0.039
		Model 3	23.450	0.012	0.395	0.993	0.063
CH_4 (CH_4/H_2)	Rigid	Model 1	2.357	10.633	0.362	0.967	0.027
		Model 2	2.436	4.381	0.420	0.978	0.025
		Model 3	3.228	1.041	0.582	0.995	0.018
	Flex	Model 1	2.747	1.487	0.551	0.985	0.026
		Model 2	3.367	0.367	0.767	0.975	0.055
		Model 3	3.940	0.353	0.749	0.950	0.095
H_2 (CH_4/H_2)	Rigid	Model 1	1.548	0.012	0.563	0.974	0.014
		Model 2	5.485	0.005	0.386	0.991	0.010
		Model 3	2.003	0.013	0.662	0.985	0.017
	Flex	Model 1	3.047	0.009	0.387	0.984	0.010
		Model 2	7.386	0.004	0.327	0.982	0.013
		Model 3	11.825	0.006	0.287	0.968	0.025

References

(1) Ungerer, P.; Collell, J.; Yiannourakou, M. Molecular Modeling of the Volumetric and Thermodynamic Properties of Kerogen: Influence of Organic Type and Maturity. *Energy & Fuels* **2015**, *29*, 91–105.

(2) Okiongbo, K. S.; Aplin, A. C.; Larter, S. R. Changes in Type II Kerogen Density as a Function of Maturity: Evidence from the Kimmeridge Clay Formation. *Energy & Fuels* **2005**, *19*, 2495–2499.

(3) Michalec, L.; Lísal, M. Molecular simulation of shale gas adsorption onto overmature type II model kerogen with control microporosity. *Molecular Physics* **2017**, *115*, 1086–1103.
